# Needs and fears of young people presenting at accident and emergency department following an act of self-harm: secondary analysis of qualitative data

**DOI:** 10.1192/bjp.bp.113.141242

**Published:** 2016-03

**Authors:** Christabel Owens, Lorraine Hansford, Siobhan Sharkey, Tamsin Ford

**Affiliations:** **Christabel Owens**, PhD, Lorraine Hansford, MSc, University of Exeter Medical School, Exeter, UK; **Siobhan Sharkey**, PhD, RMN, Plymouth University Peninsula School of Medicine & Dentistry, Plymouth, UK; **Tamsin Ford**, PhD, MRCPsych, University of Exeter Medical School, Exeter, UK

## Abstract

**Background**

Presentation at an accident and emergency (A&E) department is a key opportunity to engage with a young person who self-harms. The needs of this vulnerable group and their fears about presenting to healthcare services, including A&E, are poorly understood.

**Aims**

To examine young people's perceptions of A&E treatment following self-harm and their views on what constitutes a positive clinical encounter.

**Method**

Secondary analysis of qualitative data from an experimental online discussion forum. Threads selected for secondary analysis represent the views of 31 young people aged 16–25 with experience of self-harm.

**Results**

Participants reported avoiding A&E whenever possible, based on their own and others' previous poor experiences. When forced to seek emergency care, they did so with feelings of shame and unworthiness. These feelings were reinforced when they received what they perceived as punitive treatment from A&E staff, perpetuating a cycle of shame, avoidance and further self-harm. Positive encounters were those in which they received ‘treatment as usual’, i.e. non-discriminatory care, delivered with kindness, which had the potential to challenge negative self-evaluation and break the cycle.

**Conclusions**

The clinical needs of young people who self-harm continue to demand urgent attention. Further hypothesis testing and trials of different models of care delivery for this vulnerable group are warranted.

Self-harm is a serious and growing problem, particularly among young people. Self-harm refers to any act with a non-fatal outcome in which an individual initiates a behaviour (such as self-cutting) or ingests a substance with the intention of causing harm to themselves.^1^ People who self-harm, especially when young, are a vulnerable but largely hidden population, who do not often come into contact with services and for whom a presentation to accident and emergency (A&E) represents a key opportunity for engagement and possible suicide prevention.^2^ This opportunity is frequently missed.^3^ A systematic review of the perceptions of people who present at A&E following an act of self-harm shows that they generally report poor experiences of care.^4^ Despite publication of National Institute for Health and Care Excellence (NICE) guidance on general hospital management of self-harm,^5^ dissatisfaction remains widespread. Patients continue to complain that professionals lack understanding, do not treat them with care and respect, and fail to communicate with them effectively or to involve them in their care. Correspondingly, staff working in A&E report negative attitudes towards people who self-harm, including feelings of irritation, anger and frustration.^6,7^

Existing research relates to adults or mixed adult/adolescent populations. The views of young people who self-harm are very difficult to access and their fears about presenting to healthcare services, including A&E, are not well understood. We re-examined an existing qualitative data-set that contained spontaneous peer-to-peer talk among a group of young people who self-harm and sheds a clear light on their perceptions of A&E services, their experiences of A&E care and their views on what constitutes a positive clinical encounter.

## Method

Secondary analysis refers to the use of existing data, either by members of the original research team or by other researchers, to answer new questions or extend the focus of the primary research. Although there is a long tradition of sharing and re-using quantitative data-sets, the practice is less well established within qualitative research, possibly due to the importance attached to first-hand knowledge of the contexts in which data are constructed, as well as concerns about confidentiality.^8^ If these challenges are overcome, re-using qualitative data can be highly profitable, as they are time-consuming and expensive to collect and typically range over topics that were not anticipated at the outset. It is particularly advantageous in research with marginalised groups, whose views may be difficult to elicit in the first place.^8,9^

The data presented here are drawn from an experimental online discussion forum which was open 24 h a day for 14 weeks during the summer of 2009. The forum was set up to bring together junior health professionals and young people who self-harm and observe their verbal behaviour and discourse. The aim of the primary study was to see whether an anonymous online environment could break down some of the reported barriers to communication between these two groups, enabling them to talk on equal terms and share learning about self-harm and its management. Young people aged 16–25 with experience of self-harm (*n* = 77) were recruited from existing online self-harm forums. Recently and nearly qualified professionals in relevant mental healthcare disciplines (*n* = 18) were recruited to take part in the study, but most did not actively participate in the forum. In their absence, the young people engaged in lively discussion, supported one another through emotional crises and built a vibrant online community of their own. Full details and results of the primary study are available elsewhere.^10–14^ Three of the present authors (C.O., S.S. and T.F.) were members of the original research team. The primary study received ethical approval from Southampton & South West Hampshire NHS Research Ethics Committee A, and the present analysis falls within the scope of the original consent.

The forum generated thousands of posted contributions and provided a wealth of insight into the lived worlds of young people who self-harm. Much of the young people's talk revolved around real-world encounters with health professionals in different settings: A&E, primary care, secondary mental health, and the voluntary and private sectors. The present study focused on the young people's experiences of seeking treatment in A&E for self-inflicted injuries, including self-poisoning.

The forum was structured in such a way that posted material fell into three broad categories: discussion/debate; ‘crisis’ posts or requests for emotional support to deal with personal difficulties; and ‘random stuff’, which included off-topic chat and games. Twenty-nine (out of 87) threads initiated by young people in the discussion/debate category dealt specifically with aspects of clinical care, under titles such as: ‘+ve/−ve A&E experiences’ and ‘The best/worst things a pro [healthcare professional] can say to you’. A further six (out of 114) ‘crisis’ threads included discussion of clinical encounters in A&E. We used an in-built search tool to search the archived forum for any remaining references to A&E visits, using a range of search terms, including accident, emergency, A&E, hospital, casualty, nurse, doctor, wound, stitch and overdose.

The data thus identified were subjected to inductive thematic analysis.^15^ Three authors (L.H., C.O. and S.S.) read and familiarised themselves with all the textual material and noted down points of interest. They met several times to compare notes and agree on a set of initial codes, which were used to sort units of data into meaningful categories. Coding and subsequent retrieval were facilitated by NVivo software (www.qsrinternational.com/products_nvivo.aspx). Thematic mapping techniques, as described by Braun & Clarke,^15^ were used at later team meetings to identify candidate themes and consider their relationships to one another, their ability to represent the whole data-set and their usefulness. Material relevant to each theme was then scrutinised closely, organised into a coherent and internally consistent account, and finally embedded within an overall narrative.

## Results

Of 77 young people who registered to take part in the forum, the views of 31 are represented in the threads selected for secondary analysis. Characteristics of the full cohort and the subsample are described in Table 1. Four main themes are presented here, which correspond to stages on the young person's journey into and through A&E, namely: influences on the decision to attend or avoid; feelings on arrival; perceptions of treatment and care, and consequences of perceived negative treatment.

**Table 1:** Characteristics of participants

Characteristic	Whole cohort of young people in primary study (*n* = 77) *n* (%)	Young people included in secondary analysis (*n* = 31) *n* (%)^a^
Mean age, years	19.3	19.5

Female	73 (95)	30 (97)

White ethnic origin	74 (96)	30 (97)

Last time self-harmed		
In last 7 days	34 (44)	17 (55)
In last month	20 (26)	7 (23)
1–6 months	17 (22)	4 (13)
7–12 months	2(3)	1 (3)
1–4 years	4 (5)	2(6)
5 or more years	–	–

Method of self-harm (not mutually exclusive)		
Cutting	77 (100)	31 (100)
Not eating	50 (65)	22 (71)
Overdosing	48 (62)	16 (51)
Burning	44 (57)	14 (45)
Biting	35 (45)	13 (42)
Misusing alcohol/drugs	35 (45)	14 (45)
Binge eating	34 (44)	13 (42)
Other (e.g. head banging, hair pulling, bruising, broken bones)	40 (52)	18 (58)

a. Percentage of subsample.

### Influences on the decision to attend or avoid

It was clear from the young people's talk that they were in the habit of treating their own self-inflicted injuries whenever possible and were adept at doing so. Attendance at A&E was regarded as a last resort and was limited to those occasions on which injuries were too severe to manage at home (for example, if bleeding could not be controlled), failed to heal or developed complications. Their own previous bad experiences of A&E care and those recounted by friends were the main reason for putting off a visit for as long as possible:

‘I've self-harmed badly today and now feel ashamed about it … I took some pills and jumped off a ledge roughly 15 feet up onto tarmac … I'm so stupid … I think I may have broken something but I really don't wanna go to hospital as they were really judgemental and impatient the last time I went and I feel rubbish enough as it is.' (ID 90)

‘I've never been to A+E. I've been scared away by all the horror stories that I've heard, so consequently I have some nasty scars from wounds that could probably have used stitches.’ (ID 61)

The first participant was eventually persuaded by a close friend to go to A&E, where it was confirmed that several bones had been broken. The decision to attend was frequently prompted by lay or, very occasionally, professional referral, as here:

‘I've been to A&E this afternoon after being pestered by my practice nurse for the last 2 weeks with her concerns over a wound.’ (ID 34)

Attendance may have been involuntary, for example, if the young person was unconscious following an overdose.

### Feelings on arrival

The predominant emotions expressed in the young people's stories of their self-harm episodes were shame and self-loathing. The sense of shame was sometimes associated with a perceived ‘failure’ to have done what they set out to do, namely to take their own life. These feelings accompanied them to hospital, so that they arrived feeling worthless and undeserving of treatment:

‘I'm usually in a state where I believe I'm worthless, having failed to have the courage to go through with it properly and not feeling worthy of living.’ (ID 59)

They also talked about feeling highly vulnerable, fearful and desperate to be shown a little kindness. Many self-harmed in secret and were unable or unwilling to ask their families for support, leading to feelings of acute isolation:

‘I've been told I have to have an operation on it in the morning. They wanted to keep me in overnight but agreed I could come home if I go back at 7.30 in the morning. I feel so scared and alone as none of my family know, which is why I couldn't stay there overnight … My aunty is having a BBQ tonight and I really don't feel strong enough to put on the front, but I have no reason for not going. Aaaahhhhh, self-harm ruins everything!!!’ (ID 91)

This excerpt illustrates the extent to which the young people were troubled by their own behaviour and hated the way in which it complicated their lives, bringing them into conflict with their families and necessitating subterfuge.

Deception also characterised their visits to A&E. Lying about the origin of the injury was one of several strategies they had for managing the stress and shame of having to ask for help for a self-inflicted wound:

‘I've had an awful week and ended up shattering my wrist against a wall … I had to lie to the hospital so they didn't think I'm stupid.’ (ID48)

### Perceptions of treatment and care

The discussion threads contained numerous stories of perceived poor treatment and negative attitudes on the part of A&E staff:

‘Some nurses … just look at you with utter disgust like you're some monster.' (ID 24)

‘I was treated from start to finish as if I was pathetic and not worthy of treatment.’ (ID 90)

Some participants complained of unfair discrimination and of having been denied usual care, including pain relief, on account of having caused their own injuries. One young person spent several hours debating with fellow forum participants whether or not to get a wound looked at and, having finally summoned the courage to do so, reported:

‘They refused to treat me!! … basically 'cos it's self-harm … I feel like giving up. What's the point if no-one even wants to try and help.’ (ID41)

The group engaged in extensive discussion of those who endanger their health in other ways, and commented that, although people who self-harm are no more irresponsible and no less deserving of medical care, they nonetheless seem to be penalised more harshly for their behaviours. There was concern that discrimination could make it difficult for them to get treatment for genuinely accidental injuries:

‘Last year I [accidentally] sliced my thumb open right down to the bone … I was almost refused treatment because of the cuts on my arms. It's really irritating! … They don't refuse to treat people who do risky sports and receive a lot of injuries through them.' (ID 53)

‘Yeah … a doctor doesn't refuse to treat someone who has liver problems through drinking or a smoker with bronchitus *[sic]*.’ (ID 80)

Others reported that, although they had received basic medical attention, they felt they had been been treated as a *persona non grata*. One complained of having been ‘stuck in an out-of-the-way cubicle and ignored’, which gave her the opportunity to continue self-harming. Others considered that they had been denied information, excluded from decision-making or were talked about as if they were not present:

‘Some doctors seem to think there is a relationship between self-harm and not being able to hear, so they don't bother addressing you but just talk to anyone who happens to be with you … [They] say things like, “when did she do this?” as if the person who did it isn't capable of answering.’ (ID80)

They described feeling belittled by hospital staff, being told that that they were ‘selfish’, ‘inconsiderate’, ‘as bad as people who make hoax ambulance calls’ and that they were ‘wasting time that could be used on *real* patients’, which only served to reinforce their negative self-image and make them feel worse than when they went in. These adverse consequences are expanded on in the next section.

There were also stories of positive encounters with A&E staff. Behaviours that were particularly valued by the young people were those that demonstrated sensitivity and a genuine desire to understand the functions of self-harm:

‘I allowed a student nurse to observe and she was really kind and asked me why I self-harm because she said she didn't really understand it, and it was really nice … to be able to actually help someone learn about it.’ (ID 24)

Other examples of good practice, as judged by the young people, included: asking before taking blood ‘because the process is triggering for some people’; not requiring them to roll up sleeves when having blood pressure taken ‘because she was sensitive to the fact that I probably didn't want to have scars showing’; asking whether the patient was comfortable with a doctor of the opposite sex; chatting with them in a relaxed way about about ‘random stuff’ as well as about their emotional well-being, and refraining from ‘asking the same old psych questions 100 times … “Are you crazy?” “Are you trying to kill yourself?” Blah, blah, blah’.

Several young people complained that they had been allowed to leave hospital without being offered a psychiatric assessment; others, like the one just cited, who had been assessed many times over, made it clear that they found the process tedious and futile, since it rarely resulted in any treatment or follow-up being offered.

Participants who had had both good and bad experiences concluded that A&E was simply ‘a lottery’, and that the level of care depended entirely on who was on duty at the time. It was clear that people were seen as more important than processes in determining whether their hospital experience was positive or negative. Some of the young people demonstrated a keen awareness of the pressures under which A&E staff were working and tried to make allowances for their negative behaviours on the grounds that practitioners are ‘only human’ and have their own emotional issues to deal with:

‘I think A&E departments can be very understaffed (I know my local A&E is) so the staff get very stressed and overworked and are prone to vent their frustration on patients sometimes.’ (ID 61)

‘I can understand their frustration at having to stitch someone up knowing that there is a possiblityof them returning the next day with a new injury or after re-opening the stitches … They are only human and have bad days just like anyone else.’ (ID91)

### Consequences of perceived negative treatment

The consequences of perceived negative attitudes and behaviours were threefold: reinforcing the feelings of shame and worthlessness with which the young people arrived; avoidance of future help-seeking, and adverse health outcomes, both mental and physical.

‘You feel so low after self-harming and being treated with contempt or anger or people walking on eggshells just makes it worse. If people would simply treat us in a business-like manner, with a touch of sympathy perhaps, it would help. I know it's frustrating treating a self-harmer, but taking the frustration out on us tends to push us further from the idea of getting support.’ (ID 59)

‘I will not go up there anymore, mainly because I feel like such a time waster, and I hate all the questions they ask you … I just want to get back home, hide under the duvet and die of shame … I've ended up with numerous infections however from not getting wounds treated.’ (ID34)

Some young people talked about being more likely to self-harm after leaving A&E because of the way it made them feel, and one described feeling like going home and ‘finishing the job’, i.e. making another, more determined attempt to take her own life. They also felt powerless to complain about poor treatment, being all too aware of wasting resources that could be used on ‘more deserving’ patients:

‘When you're that low you think you deserve bad treatment and are not able to complain.’ (ID 59)

## Discussion

**Fig. 1:**
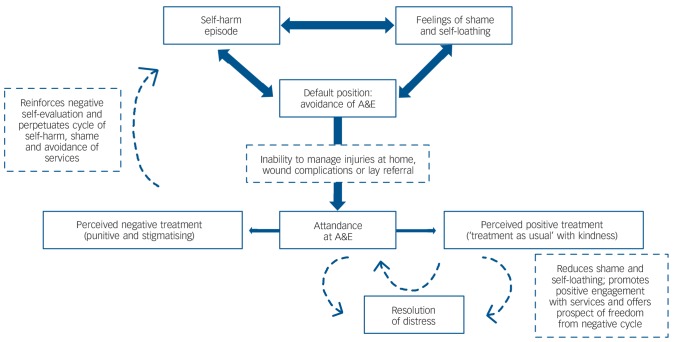
Cycle of self-harm, shame and avoidance. A&E, accident and emergency (department).

Decisions to seek treatment at A&E for self-inflicted injury are not taken lightly. Most self-harm is self-treated, and feelings of shame and unworthiness prevent young people from seeking medical help. Those negative emotions are reinforced when they encounter what they perceive to be punitive or stigmatising behaviours and a lack of empathy on the part of A&E staff, keeping them trapped in a negative cycle of shame, avoidance and further self-harm, whereas perceived positive treatment may offer hope of release from the cycle, as represented in Fig. 1.

It is nearly 35 years since publication of Jeffery's seminal paper on the ways in which A&E staff classified certain groups of patients as ‘rubbish’.^16^ ‘Rubbish’ included those who had self-harmed, whom A&E staff judged as having broken the unwritten rules of engagement with health services and as seeking illegitimate access to the sick role, and whom they therefore singled out for hostile and punitive treatment. The belief that certain A&E attenders represent ‘rubbish’ appears to be still alive and well, but in the minds of patients themselves. The young people who took part in our discussion forum evaluated themselves as ‘rubbish’ on arrival at A&E, and the slightest word or gesture on the part of a receptionist, nurse or doctor was likely to be interpreted as confirmation of that self-assessment, leaving them feeling even more worthless than when they went in and trapped in a negative spiral.

Hunter *et al* noted that psychosocial assessment following self-harm ‘had the power to reinforce or challenge hopelessness and negative self-evaluations’, as well as to encourage or discourage engagement with services.^17^ Our findings suggest that the same may be true of the visit to A&E as a whole. The fact that young people who have self-harmed arrive at A&E feeling like ‘rubbish’ may predispose them to perceive staff attitudes and behaviours as hostile and punitive, even when staff do not intend them as such. The young person who complained that she was treated as if she was ‘pathetic and not worthy of treatment’ may unwittingly have been describing her own assessment of herself. This negative view of self, together with the general emotional turmoil that those who have self-harmed bring to the situation, means that they are likely to interpret being asked to wait ‘in an out of the way cubicle’ as being shunned or stigmatised, even when no such slight is intended. Indeed, staff may believe that they are being considerate by affording the young person privacy, as recommended by NICE guidance.^5^ This underlines the acute need for open communication and involvement at all stages of treatment.^4,17^

A visit to A&E for a self-inflicted injury or overdose is a complex human encounter, with both manifest and hidden elements. The person presents with a manifest physical health need, e.g. a cut that requires stitching, but, unlike the victim of an accident, they arrive feeling contemptible, distrustful and defensive, and they arouse difficult emotional reactions and defended practice in those treating them. If those negative emotions are not brought into the open and addressed, the encounter is likely to go wrong, with adverse consequences for both parties. In a study of psychiatric nurses' interactions with patients who are suicidal, Tzeng *et al* found that nurses who were unable to appreciate patients' inner worlds distanced themselves, labelled patients as ‘attention seekers’, ‘time wasters’ or ‘nuisances’, and avoided contact with them. The patients then perceived nurses as uncaring, and both parties felt hurt and devalued by the encounter, but when nurses were willing to embrace patients' experiences and learn from them, ‘they changed not only their attitudes but also the nurse–patient relationship … from mutual hostility to “win–win” outcomes’.^18^ This message is echoed by Ballatt & Campling, who call for the notion of ‘kinship’ to be placed at the centre of healthcare.^19^ Related etymologically to kindness, kinship draws attention to the shared humanity and interconnection between clinicians and patients. Without recognition of kinship, care and compassion can easily be replaced by contempt.

Chapman & Martin report that A&E staff find those presenting with self-inflicted injuries ‘harder work’ than acutely ill patients, and ‘very time consuming’.^7^ Our findings may offer some comfort to A&E staff. Although they point to a need for clinicians to be alert to the hidden aspects of the encounter, it is clear that the young people in our study recognised the pressures on A&E staff and did not expect any special treatment. On the contrary, a positive clinical encounter, in their view, was one in which they received ‘treatment as usual’, i.e. the same level of physical care that would be offered to any other patient, delivered with the same level of openness, warmth and respect. Like any patient who finds themselves in A&E, they desired a measure of ‘sympathy’, which involves nothing more complex than an acknowledgement of their fragile emotional state, and reassurance that they are not viewed as time wasters or attention seekers. They also greatly appreciate any opportunity to help educate health professionals about self-harm.

### Strengths and limitations of the study

These findings reinforce those from studies of adults and mixed populations regarding patients' experiences of care. However, our data offer direct insight into the lifeworlds of young people who self-harm, whose voices often go unheard. This group is very hard to reach using traditional research methods, especially when recruitment is via A&E departments, where response rates as low as 6% have been reported.^17^ The young person who described wanting to ‘go home, hide under the duvet and die of shame’ after being treated in A&E is unlikely to have responded to an invitation by a member of A&E staff to take part in research, suggesting that alternative recruitment methods may need to be developed for this group. The nature of our primary study was different from standard interview or focus group studies, insofar as it explicitly offered young people who self-harm an opportunity to enter into a collaborative relationship with healthcare professionals, based on a presumption of psychological equality, and to contribute to professional education about self-harm and its management.^10^

A further strength of this data-set is that the participants were not specifically asked about their experiences of A&E. These data were unsolicited, but were produced spontaneously during the course of online discussion in participant-led threads, which continued over successive days and weeks, thus reflecting the importance of this issue for them.

Unfortunately, the non-participation of healthcare professionals in the discussion forum means that we cannot compare their perspectives with those of the young people. The discussion might have proceeded along different lines had the health professionals been present, as was originally envisaged.

The disinhibiting nature of online environments and the fact that the young people were chatting among themselves rather than participating in a formal interview may have encouraged them to exaggerate and tell ‘tall tales’ of uncaring treatment. However, the fact that their perceptions tally with those reported elsewhere, both by service users^4,17^ and by A&E staff,^6^ suggests that they are a true reflection of the way in which the young people experienced A&E care.

### Implications for research and service development

As Fig. 1 indicates, we hypothesise that positive encounters in A&E have the potential to reduce shame and challenge negative self-evaluation, encourage future help-seeking and thus contribute in the longer term to resolution of distress. This could be tested empirically.

Front-line A&E staff are often very junior and may lack knowledge about self-harm and how to respond to it. A brief training programme, emphasising the feelings of shame, self-disgust and worthlessness experienced by people who self-harm might increase understanding, reduce frustration and prompt more compassionate responses. Opportunities should be created for those who self-harm to contribute to training programmes, as this has the potential to enhance their self-esteem. This too requires empirical testing.

Trials of different models of care for those who have self-harmed may also be warranted. In a study of homeless people presenting at an emergency department, another group that are commonly viewed by staff as ‘difficult’, half were randomised to receive special attention by a volunteer, who gave them food, chatted with them and listened attentively to their concerns.^20^ The findings suggested that this led to improved patient satisfaction and a reduction in the number of return visits, thus refuting the widely-held belief that improving patient experience will lead to increased demand and cause healthcare systems, and those who work in them, to collapse under the strain. This study of ‘compassionate’ as compared with conventional care may warrant replication with those who present with self-inflicted injuries. Careful attention would need to be given to outcomes, in order to tease out whether a reduction in the number of repeat visits to A&E signified further disenchantment and avoidance (consistent with the present findings) or an improvement in health and well-being; after all, a visit to A&E may represent a life saved.^21^ There is still insufficient evidence regarding ‘caring effects’^22^ and the benefits (as opposed to the presumed risks) of empathy.^23^

‘Inappropriate attendance’ at A&E departments has long been a subject of debate, and some authors have questioned whether it is the service or the patient that is ‘inappropriate’.^24,25^ A busy emergency department that is designed to deal with acute illness and physical trauma may not be the right place to engage with those in emotional turmoil. A carefully conceived ‘sanctuary’, where they could receive support from peers or volunteers and calm themselves while waiting for treatment of injuries and/or assessment by the psychiatric team, might take some of the pressure off A&E staff, as well as helping to change attitudes. Such safe havens are already available for groups whose emotional turmoil is regarded as legitimate, such as parents who experience stillbirth.

The clinical needs and fears of those who, at whatever age and for whatever reason, are driven to self-harm continue to demand urgent attention.
